# Prosthetic Hip Dislocations After Total Hip Replacement: A Retrospective Study

**DOI:** 10.7759/cureus.83059

**Published:** 2025-04-27

**Authors:** Bratati Bandyopadhyay, Sunandan Datta, Mohammed Hussain, Muhammad Raza, Maham Mansoor, Rachala Madhu, Sudipta Kar

**Affiliations:** 1 Trauma and Orthopedics, Aneurin Bevan University Health Board, Newport, GBR

**Keywords:** posterior dislocation of hip, total hip arthroplasty dislocation, total hip athroplasty, total hip replacement (thr), traumatic posterior hip dislocation

## Abstract

Introduction

Total hip replacement (THR) dislocation occurs when the prosthetic joint components completely separate, indicating a failure in the intended mechanics of the hip joint established by the prosthesis. This complication poses significant challenges for patients and surgeons and financially burdens healthcare systems.

Methods

This retrospective observational study evaluated cases of prosthetic hip dislocation within our health board that required manipulation under anesthesia (MUA) from January 1, 2021, to March 5, 2024. A total of 60 patients met the inclusion criteria. Patient data were collected from hospital records using dedicated clinical software platforms.

Results

The patients were divided into two groups based on the treatment received: MUA and revision THR. The Mann-Whitney U test showed no significant difference in the time to dislocation between the groups, but there was a notable difference in the number of dislocations. Posterior dislocations were the most common, occurring in 36 patients (60%). Of the 60 dislocated hips, 22 (36.67%) required revision surgery, while the remaining 38 cases were managed with MUA. The main cause of dislocation was noncompliance with recommended hip positioning.

Conclusions

Prosthetic hip dislocation remains a challenging complication following THR. Our analysis underscores the importance of monitoring for late dislocations and emphasizes the need for thorough patient education and adherence to postoperative precautions. Future multicenter studies are needed to validate our findings and develop standardized protocols to reduce dislocation risks.

## Introduction

Total hip replacement (THR) dislocation is defined as the complete separation of prosthetic joint components, indicating a failure of the mechanical function intended by the prosthesis [1]. This complication presents substantial challenges for both patients and surgeons and imposes a considerable financial burden on healthcare systems [2]. The incidence of post-THR dislocation is influenced by multiple factors, including surgical technique, patient-specific characteristics, and the type of implant used. Studies conducted at major institutions report dislocation rates ranging from 0.3% to 3% following primary THR for osteoarthritis [3]. In comparison, revision procedures and implant exchanges demonstrate significantly higher dislocation rates, reaching up to 28% [4].

Dislocation after THR has profound implications, contributing to both physical and emotional distress and substantially reducing patients’ quality of life. Furthermore, it is one of the leading causes of early revision surgeries [5,6].

The primary objective of this study was to analyze cases of prosthetic hip dislocation within our health board that required manipulation under anesthesia (MUA) following both primary and revision THR. Our study also takes a look at the difference in dislocation episodes and timing following both primary and revision THR. Additionally, this study aimed to assess the proportion of these cases that necessitated revision surgery as a definitive intervention and to identify the most common causes of dislocation in order to develop effective prevention strategies.

## Materials and methods

This retrospective observational study was conducted as an audit to evaluate cases of prosthetic hip dislocation within our health board requiring MUA. The study covered the period from January 1, 2021, to March 5, 2024, identifying 60 patients who met the inclusion criteria.

Patient data were obtained from electronic hospital records using Clinical Workstation, Ormis, and the Welsh Clinical Portal, which provided detailed information on patient presentations, surgical histories, and procedures.

Inclusion criteria were prosthetic hip dislocation following THR managed with MUA during either an emergency department visit or inpatient stay. Exclusion criteria included dislocations after hip hemiarthroplasty, native hip dislocations, and cases with incomplete data.

Study population

A total of 60 consecutive patients with prosthetic hip dislocation requiring MUA were included. Of these, 42 were male and 18 were female.

Data analysis

Statistical analysis was performed using IBM SPSS Statistics for Windows, Version 22.0 (Released 2013; IBM Corp., Armonk, NY, USA). Continuous variables were summarized with means, medians, and IQRs; categorical variables were expressed as percentages. Mann-Whitney U and Kruskal-Wallis tests were used to assess nonparametric data. Categorical variables were compared using the chi-square test, and continuous variables using the one-way ANOVA test. Logistic regression analyzed associations with dislocation outcomes.

## Results

The study population was divided into two groups based on definitive treatment: MUA or revision THR. A total of 60 patients experienced prosthetic hip dislocation requiring MUA. Of these, 48 dislocations followed primary THR, and 12 occurred after revision procedures. The mean patient age was 66.5 years, with a female predominance (42 females and 18 males). No significant sex-based differences in outcomes were observed.

Dislocation characteristics

**Figure 1:**
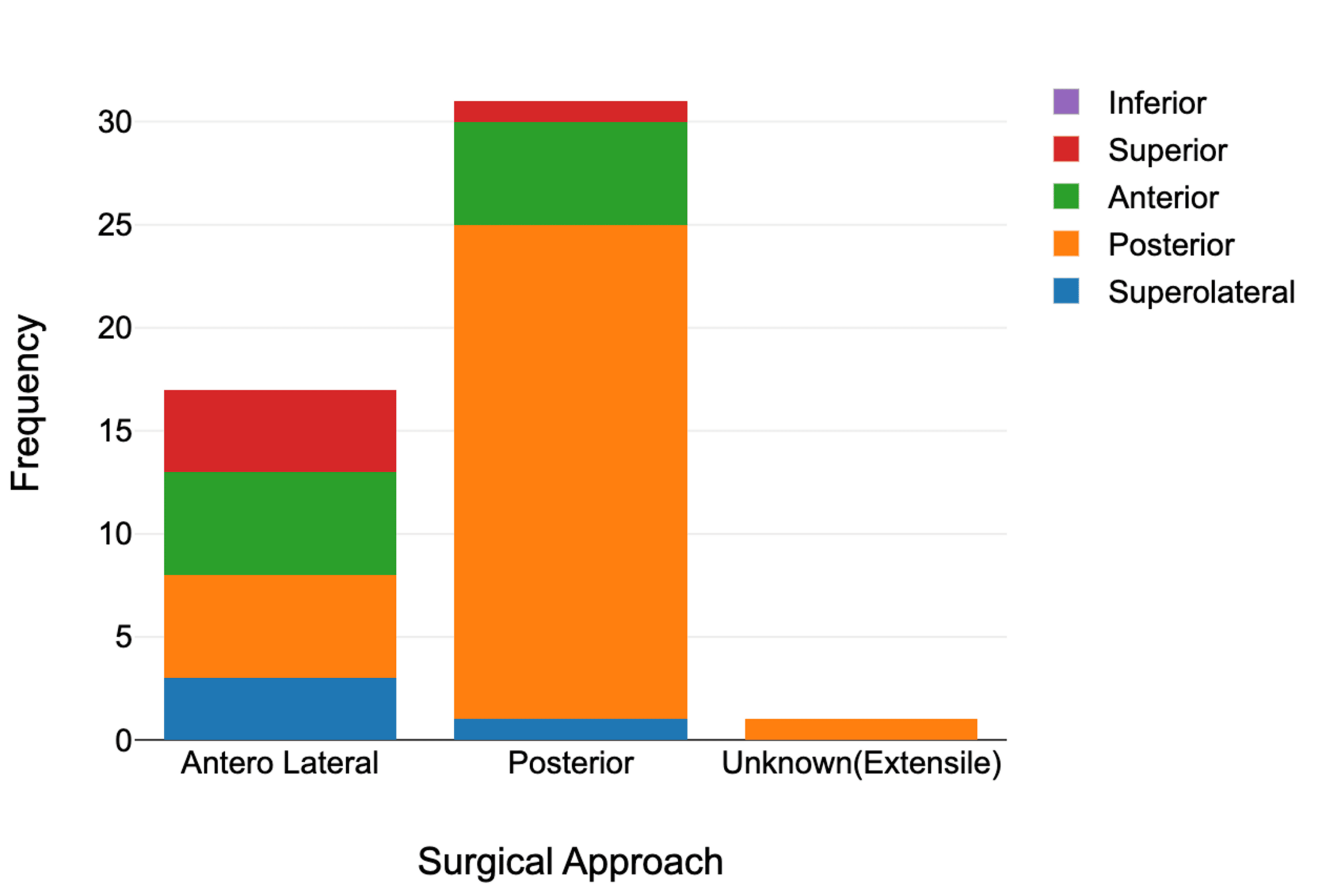
Proportion and frequency of dislocation types by surgical approach A stacked bar chart illustrates both the proportion and frequency of prosthetic hip dislocations, categorized by dislocation type and surgical approach. Posterior dislocations were the most common, predominantly following the posterior approach. Fewer cases of anterior, superior, and superolateral dislocations were observed across all approaches. Dislocation types are color-coded as follows: green for anterior, orange for posterior, red for superior, and blue for superolateral.

Posterior dislocations were the most common, occurring in 37 cases (61.67%), followed by anterior in 11 cases (18.33%), superior in six cases (10%), superolateral in five cases (8.33%), and inferior dislocation in one case (1.67%). The posterior surgical approach was most frequently documented (32 cases), followed by anterolateral (17 cases). In 11 cases, the approach was unspecified, and in one case, the approach was mentioned extensile approach without any more details. There was no statistically significant association between surgical approach and dislocation type (Table 1, Figure 1). A chi-square test was used to evaluate the association between surgical approach and type of dislocation, both categorical variables. A one-way ANOVA compared the mean number of dislocations, a continuous variable, across different surgical approach groups.

**Table 1:** Comparison of surgical approach, dominant type of dislocation, and average number of dislocations This table compares surgical approaches in relation to the dominant type of dislocation, the average number of dislocations, and the statistical significance of these associations. Statistical analysis involved chi-square testing for categorical variables (dislocation type vs. surgical approach) and one-way ANOVA to compare the mean number of dislocations across groups. A statistically significant association was found between surgical approach and type of dislocation (p = 0.044), while no significant difference was observed in the average number of dislocations between groups (p = 0.744).

Surgical approach	Most common type of dislocation (count)	Average number of dislocations	Chi-square value	F statistic	Chi-square p-value	ANOVA p-value
Anterolateral	Anterior (5)	2.118	12.961	0.298	0.044	0.744
Posterior	Posterior (24)	2				
Unknown (extensile)	Posterior (1)	1				

The types of prostheses varied: 28 hips had uncemented implants, 10 were cemented, and 22 were hybrids. Dislocations were most commonly associated with femoral head sizes of 28 mm and 36 mm, while the 32 mm head size was associated with the fewest dislocations.

Dislocation episodes and timing 

Of the 60 patients, 30 had a single dislocation episode, 12 had two, seven had three, and two patients experienced six and seven episodes, respectively. The mean time from index surgery to first dislocation was 69.3 months. The average age at first dislocation in the MUA group was 64.9 years (SD: 15.5), compared to 69.2 years (SD: 11.5) in the revision group. The mean time to first dislocation was 70.7 months in the MUA group and 67.0 months in the revision group. The average number of dislocations was 1.79 in the MUA group versus 2.95 in the revision group. The Mann-Whitney U test revealed no significant difference in the time to dislocation between primary and revision THR groups (p = 0.91); however, there was a statistically significant difference in the number of dislocations between the two groups (p = 0.004), as illustrated in Table 2 and Table 3.

**Table 2:** Comparison of MUA and THR revision groups for time to dislocation (in months) This table compares the MUA group and the THR revision group in terms of time to the first dislocation. The mean time to dislocation was 70.67 months for the MUA group and 67.04 months for the THR revision group. However, this difference was not statistically significant (p = 0.91) based on the Mann-Whitney U test (U value = 381). MUA, manipulation under anesthesia; THR, total hip replacement

Procedure	N (total number)	Median (months)	Mean (months)	SD (months)	Test statistic (U value)	p-value
MUA	38	24	70.66964865	89.65504502	381	0.909
THR revision	22	36	67.04114286	84.39297966		

**Table 3:** Comparison of MUA and THR revision groups for number of dislocations This table compares the MUA group and the THR revision group in terms of the number of dislocations. The mean number of dislocations was 1.79 for the MUA group and 2.95 for the THR revision group. This difference was found to be statistically significant, as indicated by the Mann-Whitney U test (p-value = 0.0035). MUA, manipulation under anesthesia; THR, total hip replacement

Procedure	N (total number)	Median	Mean	SD	Test statistic (U value)	p-value
MUA	38	1	1.789474	1.473329	240.5	0.00351
THR revision	22	2.5	2.954545	1.758714		

**Figure 2:**
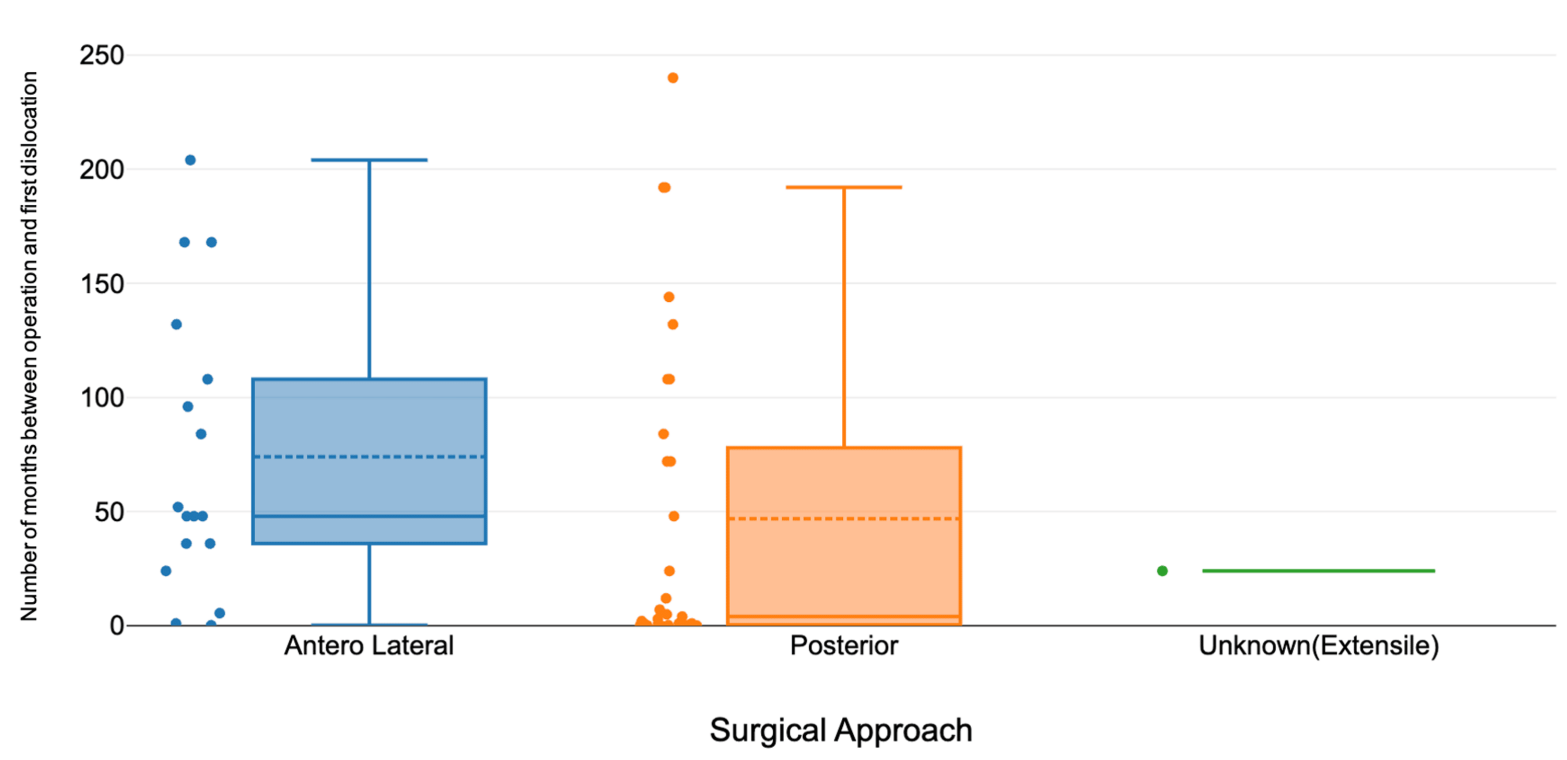
Time to dislocation (from operation to first dislocation) in months according to surgical approach A boxplot comparing the time interval (in months) between index surgery and the first dislocation across different surgical approaches in patients undergoing THR. The anterolateral approach shows a broader IQR and a higher median time to dislocation compared to the posterior approach, suggesting greater variability and potentially a later onset of instability. Several outliers are observed in both groups, indicating cases of delayed dislocation. Dashed lines represent group means, while solid lines within the boxes indicate medians. THR, total hip replacement

Revision surgery was required in 22 cases (36.7%), while 38 patients (63.3%) were managed successfully with MUA. The Kruskal-Wallis test showed no statistically significant differences in time to first dislocation (H statistic: 4.582, p-value: 0.101) or number of dislocations (H statistic: 0.812, p-value: 0.666) across surgical approaches (Figure 2). Noncompliance with hip precautions was the most common cause of dislocation, followed by trauma and excessive range of motion.

## Discussion

Dislocation is a leading cause of revision THR in the United States and ranks fourth in the United Kingdom [7,8]. Aseptic loosening is the most common cause of revision THR worldwide, followed by dislocation [9].

Dislocation risk factors can be categorized as patient specific, procedure specific, or implant related. Patient-related factors include advanced age, neurological conditions, and poor adherence to postoperative care [10,11]. Procedure-specific risks involve surgical approach, component positioning, soft tissue tension, and surgeon experience [12,13]. Implant-related factors - such as small head-neck ratios, over-hemispheric acetabular cups, and extended prosthetic heads - may predispose patients to impingement and subsequent dislocation. While larger femoral heads (≥36 mm) improve range of motion and stability, they may also cause secondary impingement between the femoral neck and pelvic bone. In our cohort, 28 mm and 36 mm heads were most frequently associated with dislocations [14,15].

Consistent with Christensen et al., who reported that 68% of hip dislocations were posterior [16], our study found that posterior dislocations comprised 61.67% of cases. Woolson and Rahimtoola reported a 3.2% dislocation rate across 10,500 THRs [17]. Notably, dislocation rates are higher after revision THR, with some studies reporting rates as high as 25% [18].

Dislocation timing is a key consideration. Fessy et al. observed that over 50% of dislocations occur within three months postoperatively [19], while Ali Khan et al. reported that 66% occurred within five weeks [20]. In contrast, our data showed only 20.7% of dislocations occurred within the first five weeks, possibly reflecting improvements in early rehabilitation and patient education. Late dislocations accounted for 36.2% of our cases. These are often attributed to polyethylene wear. Huten and Langlais estimated that 32% of dislocations occurred on average 11.3 years post-THR [21]. In our study, the mean time to late dislocation was 14.2 years, emphasizing the need for long-term follow-up.

Although Taunton et al. reported that the posterior approach was associated with the highest dislocation rates after primary THR [22], our study did not find a statistically significant association between surgical approach and number of dislocations. This suggests that patient factors and implant design may play a more prominent role. Our cohort had a mean of 2.22 dislocations per patient, consistent with the 2.81 reported by Blom et al. [23], underscoring the complexity of managing recurrent hip dislocations and the need for individualized treatment strategies.

Our study has several limitations due to its retrospective design. The modest sample size (n = 60) reduces statistical power and generalizability. Data extracted from multiple clinical systems may impact accuracy, depending on documentation consistency. Missing surgical approach data in 11 cases introduces potential bias, while unexamined factors such as implant positioning, surgeon-specific variables, patient comorbidities, rehabilitation compliance, and socioeconomic factors further limit the study’s conclusions on dislocation outcomes.

## Conclusions

Prosthetic hip dislocation remains a challenging complication following both primary and revision THR surgeries. Our retrospective analysis of 60 patients reveals several important insights for future management and prevention. It was observed that the average number of dislocations was higher in the revision THR group as compared to the MUA group. Posterior dislocations were the most prevalent (60%), consistent with literature linking the posterior approach to greater instability; however, no statistically significant association was identified between the surgical approach and number of dislocations in our cohort. These findings reinforce the multifactorial nature of dislocation risk. Patient-related factors were frequently implicated, with noncompliance with postoperative precautions identified as the leading cause of early dislocation, followed by trauma and excessive range of motion. These findings support the critical role of patient education, structured rehabilitation, and adherence to movement restrictions in mitigating dislocation risk in the first few months after surgery. Discharge planning should include reinforcement of postoperative instructions and the provision of assistive devices where appropriate. Future multicenter studies are warranted to validate our findings and support the development of standardized protocols to minimize dislocation risk.
